# Helicopter Emergency Medical Services (HEMS) response to out-of-hospital cardiac arrest

**DOI:** 10.1186/1757-7241-21-S1-S9

**Published:** 2013-05-28

**Authors:** RM Lyon, MJ Nelson

**Affiliations:** 1 Kent, Surrey, Sussex Air Ambulance, UK

## Background

Out-of-cardiac arrest (OHCA) is a common medical emergency with significant mortality and significant neurological morbidity. Helicopter emergency medical services (HEMS) may be tasked to OHCA. We sought to assess the impact of tasking a HEMS service to OHCA and characterise the nature of these calls.

## Method

Retrospective case review of all HEMS calls to Surrey and Sussex Air Ambulance, United Kingdom, over a 1-year period (1/9/2010-1/9/2011).

## Results

HEMS was activated 89 times to suspected OHCA. This represented 11% of the total HEMS missions. In 23 cases HEMS was stood-down en-route and in 2 cases the patient had not suffered an OHCA on arrival of HEMS. 25 patients achieved return-of-spontaneous circulation (ROSC), 13 (52%) prior to HEMS arrival. The HEMS team were never first on-scene. The median time from first collapse to HEMS arrival was 31 minutes (IQR 22-40). The median time from HEMS activation to arrival on scene was17 minutes (IQR 11.5-21). 19 patients underwent pre-hospital anaesthesia, 5 patients had electrical or chemical cardioversion and 19 patients had therapeutic hypothermia initiated by HEMS. Only 1 post-OHCA patient was transported to hospital by air.

## Conclusion

OHCA represents a significant proportion of HEMS call outs. HEMS most commonly attend post-ROSC OHCA patients and interventions, including pre-hospital anaesthesia and therapeutic hypothermia should be targeted to this phase. HEMS are rarely first on-scene and should only be tasked as a first response to OHCA in remote locations. HEMS may be most appropriately utilised in OHCA by only attending the scene if a patient achieves ROSC.

